# Automated Measurements of Long Leg Radiographs in Pediatric Patients: A Pilot Study to Evaluate an Artificial Intelligence-Based Algorithm

**DOI:** 10.3390/children11101182

**Published:** 2024-09-27

**Authors:** Thies J. N. van der Lelij, Willem Grootjans, Kevin J. Braamhaar, Pieter Bas de Witte

**Affiliations:** 1 Department of Orthopaedics, Leiden University Medical Center, 2333 ZA Leiden, The Netherlands; t.j.n.van_der_lelij@lumc.nl (T.J.N.v.d.L.);; 2 Department of Radiology, Leiden University Medical Center, 2333 ZA Leiden, The Netherlands; w.grootjans@lumc.nl

**Keywords:** artificial intelligence, leg angle measurement assistant, LAMA, long leg radiographs, pediatric, orthopedics

## Abstract

Background: Assessment of long leg radiographs (LLRs) in pediatric orthopedic patients is an important but time-consuming routine task for clinicians. The goal of this study was to evaluate the performance of artificial intelligence (AI)-based leg angle measurement assistant software (LAMA) in measuring LLRs in pediatric patients, compared to traditional manual measurements. Methods: Eligible patients, aged 11 to 18 years old, referred for LLR between January and March 2022 were included. The study comprised 29 patients (58 legs, 377 measurements). The femur length, tibia length, full leg length (FLL), leg length discrepancy (LLD), hip–knee–ankle angle (HKA), mechanical lateral distal femoral angle (mLDFA), and mechanical medial proximal tibial angle (mMPTA) were measured automatically using LAMA and compared to manual measurements of a senior pediatric orthopedic surgeon and an advanced practitioner in radiography. Results: Correct landmark placement with AI was achieved in 76% of the cases for LLD measurements, 88% for FLL and femur length, 91% for mLDFA, 97% for HKA, 98% for mMPTA, and 100% for tibia length. Intraclass correlation coefficients (ICCs) indicated moderate to excellent agreement between AI and manual measurements, ranging from 0.73 (95% confidence interval (CI): 0.54 to 0.84) to 1.00 (95%CI: 1.00 to 1.00). Conclusion: In cases of correct landmark placement, AI-based algorithm measurements on LLRs of pediatric patients showed high agreement with manual measurements.

## 1. Introduction

Long leg radiographs (LLRs) serve as a crucial diagnostic tool for assessing bone length, lower limb alignment, and joint line orientation. Specifically in children, LLRs play an important role in the diagnosis and quantification of various limb malalignments and deformities, including genu varum (bow legs), genu valgum (knock knees), and leg length discrepancy (LLD) [1]. However, performing and interpreting length and angle measurements manually on LLRs of patients is very time-consuming for clinicians and prone to intra-and interobserver bias [2,3,4]. In this study, we evaluated the performance of an artificial intelligence (AI)-based software application for automatic assessment of LLRs in pediatric patients.

LLD and lower limb malalignment are common pediatric orthopedic issues that are associated with various musculoskeletal disorders including gait deviation, scoliosis, low back pain, osteoarthritis, and compromised postural control [5,6]. Although an LLD < 1 cm is often asymptomatic and present in up to 90% of the population, LLD in children can be progressive, and LLD > 2 cm may become symptomatic later in life [6,7,8,9]. Similarly, malalignment (e.g., valgus and varus leg angles) developed during childhood may increase the risk of early osteoarthritis in adulthood [10]. Minimally invasive procedures (i.e., guided growth procedures) are available to manage LLD and lower limb malalignment to prevent future symptoms [8]. LLR measurements play a critical role in the clinical decision-making and follow-up for children treated with guided growth procedures [11,12,13,14]. Automation of such measurements with the use of artificial intelligence (AI) techniques, particularly deep learning (DL) algorithms, has the potential to improve the speed, accuracy, and efficiency of these evaluations. This could save time for clinicians and subsequently for patients while at the same time improving the consistency and accuracy of LLR measurements [15,16,17].

Recent studies have investigated the use, performance, and added value of AI-based algorithms in orthopedic radiology [15]. However, little is known about AI-based measurement programs for LLR in children. We set out to explore an AI-based leg angle measurement assistant (LAMA) that can automate length and angle measurements on LLRs. In previous studies, the performance of this AI-based algorithm has been studied in adults [16,17,18,19,20]. To our knowledge, there are no studies that have used the LAMA software to evaluate a comprehensive set of LLR measurements, including bone length and joint angle measurements, specifically in the pediatric population. The latter is actually one of the largest groups of patients undergoing these radiologic investigations. If the measurements of the AI-based algorithm are consistent with manual measurements, the software may be used in clinical practice as an adjunct or even substitute for the traditional manual measurements, thereby saving valuable time for both clinicians and patients. 

Therefore, the aim of this study was to assess the accuracy and reliability of measurements performed by the LAMA software compared to manual measurements on LLRs in patients under the age of 18 years. 

## 2. Materials and Methods

### 2.1. Patient Inclusion and Image Acquisition

For this observational cohort study, the study population consisted of pediatric patients referred for LLR between January and March 2022. Patients were included if they were aged 11 to 18 years at the time of LLR. The following exclusion criteria were used: visual artifacts or poor visibility on radiographs, incorrect positioning, non-weight-bearing or abnormal cropping. Abnormal cropping refers to the issue where an LLR does not capture the entire area of the leg(s) or where part of the leg(s) is unintentionally excluded from the radiograph. This can occur due to incorrect patient positioning, improper image capture settings, or technical errors during the imaging process. Artifacts were defined by abnormal or misleading image features that were not caused by the patient’s anatomy. LLRs were acquired in a standardized, weight-bearing manner using a digital Aseco+ X-ray system with CXDI detectors (Canon Medical Systems Corporation, Otawara, Japan). The imaging parameters included a tube voltage of 85 kVp and a tube current of 450 mA. Three separate X-ray images were taken: (1) pelvis to mid-femur, (2) mid-femur to mid-tibia, and (3) mid-tibia to foot. These images were subsequently stitched together to create a single LLR image.

### 2.2. Measurements

All measurements on the LLRs were performed by two observers and LAMA. Firstly, a senior pediatric orthopedic surgeon (>5 years of experience) and an advanced practitioner in radiography (>15 years of experience) independently performed all measurements manually. Observers were blinded to each other’s measurements. Secondly, automatic assessment of LLRs was performed using commercially available software based on DL technology (LAMA, version 1.03, ImageBiopsy Lab, Vienna, Austria). The LAMA application was trained on a dataset comprising over 15,000 radiographs sourced from various studies, including the Osteoarthritis Initiative, the Multicenter Osteoarthritis Study, the Cohort Hip and Cohort Knee study, and five sites in Austria [21,22,23]. The training cohort contained LLRs from adult patients of different ages and ethnic backgrounds and data acquired using different radiography systems. In order to perform the measurements, the LAMA application identifies anatomical bony landmarks and provides measurements of angles and lengths. For additional details on the model training, readers are directed to the supplement provided by Simon et al. [17].

### 2.3. Image Analysis

**Figure 1:**
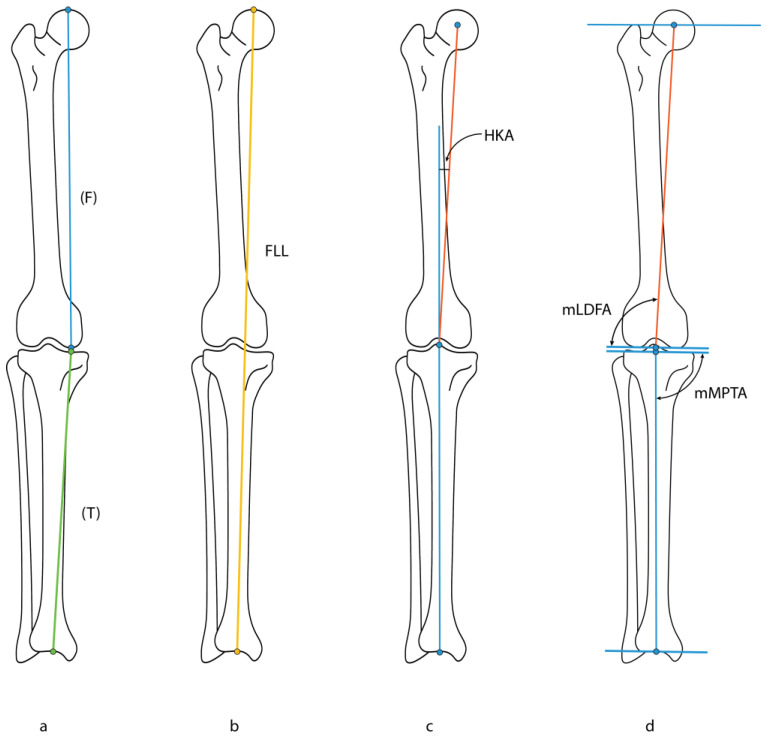
Schematic overview of measurements performed on long leg radiographs (LRRs), including (**a**) femur length (F) and tibia length (T), (**b**) full leg length (FLL), (**c**) hip–knee–ankle angle (HKA), (**d**) mechanical lateral distal femoral angle (mLDFA), and mechanical medial proximal tibial angle (mMPTA).

Manual assessment of LLRs was performed using IDS7 software (Sectra AB, IDS7 version 25.2, Linköping, Sweden). With regard to automated measurements, results of the LAMA analysis were visually evaluated in the IDS7 software to assess the correct placement of landmarks, including the top of the femoral head, the medial femoral condyle, the mid-tibial roof, the mechanical axis of the tibia, the proximal tibial knee joint orientation line, the mechanical axis of the femur, and the distal femoral knee joint orientation line (Table 1). With these landmarks, the following measurements were obtained: femur length, tibia length, full leg length (FLL), leg length discrepancy (LLD), hip–knee–ankle angle (HKA), mechanical lateral distal femoral angle (mLDFA), and mechanical medial proximal tibial angle (mMPTA) (Figure 1). Cases with incorrect measurements due to the inability of LAMA to identify the correct landmarks were excluded. 

### 2.4. Statistical Analyses

Patient demographics and distributions of all measurements (2 observers) were evaluated using descriptive statistics: means with standard deviations (in case of normally distributed data) and medians with ranges (in case data were not normally distributed). The primary outcome was correct landmark placement by LAMA. The secondary outcome of this study was the comparison of quantitative analyses (i.e., agreement) between LAMA and manually performed measurements. The manually performed measurements of 2 observers were compared to each other and to the LAMA results. 

Paired *t*-tests were used to compare the mean of manual measurements (2 observers) to the measurements obtained by using LAMA for each of the 29 patients (total of 58 legs). For analysis of LLD, left and right legs were compared. Analysis of interobserver agreement was performed by comparing manual measurements between the two observers. Agreement was determined by calculating the intraclass correlation coefficients (ICCs) [24]. We assessed the agreement between the observers using an absolute agreement ICC in a two-way random effects model [25]. Furthermore, agreement was determined between the mean values of the manual measurements (of the two observers) and measurements obtained by LAMA. The agreement between the manual and AI measurements was assessed using an absolute agreement ICC in a two-way mixed effects model [25]. The categorization used for interpreting the ICC values were as follows: values less than 0.50—poor reliability, between 0.50 and 0.75—moderate reliability, between 0.75 and 0.90—good reliability, and greater than 0.90—excellent reliability [25]. Furthermore, manual and LAMA measurements were visually presented using Bland–Altman plots, including the lower and upper limits of the 95% confidence interval (CI) of agreement [26]. Analyses were performed using SPSS (version 29.0, IBM SPSS Statistics, Armonk, NY, USA).

## 3. Results

**Figure 2:**
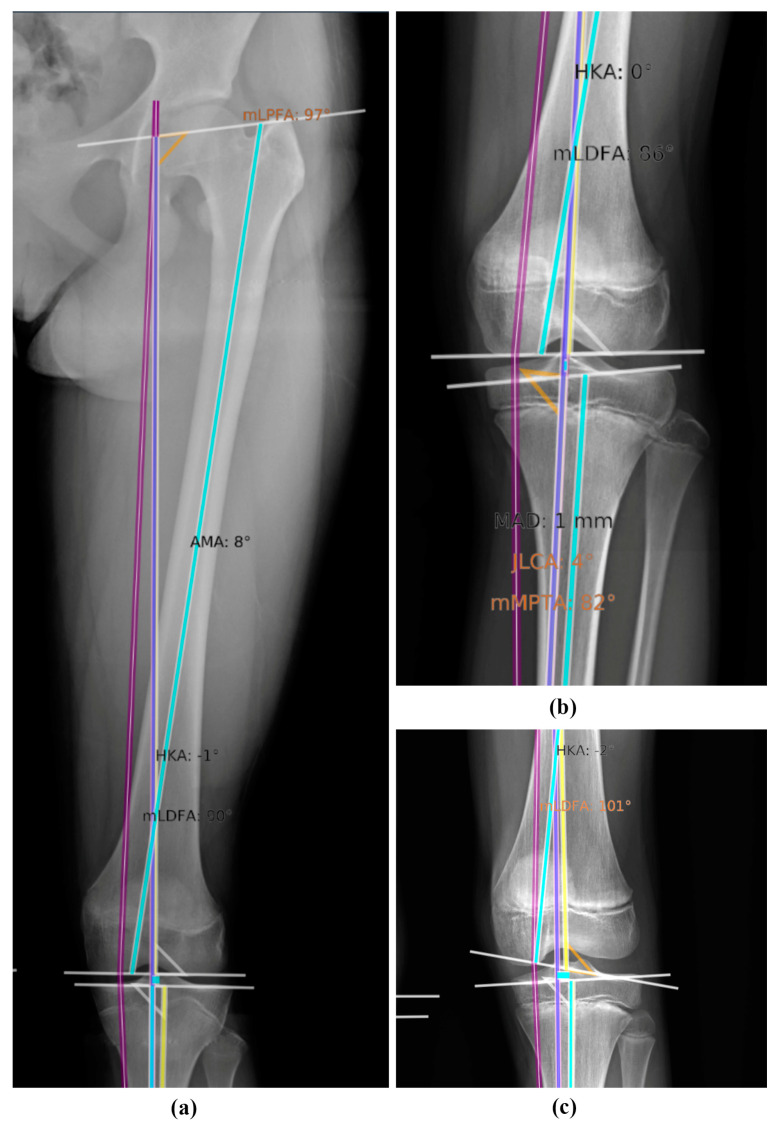
Examples of erroneously placed landmarks by the leg angle measurement assistant (LAMA) showing incorrect identification of (**a**) the top of the femoral head and femoral head center, (**b**) the proximal tibial knee joint orientation line, (**c**) the distal femoral knee joint orientation line.

A total of 29 patients (58 legs) were included in this study. The median age of the patients was 13.7 years (range 12–16). Furthermore, a total of 12 patients were male (41%) and 17 were female (59%). LLRs were taken for (suspected) LLD (12 patients), tall stature (11 patients), screening for fibrous dysplasia (1 patient), genu valgum (3 patients), or genu varum (2 patients). In a total of eight LLRs, there was erroneous placement of the anatomical landmarks by LAMA. Figure 2 demonstrates a number of these erroneously placed landmarks.

The erroneous placements were due to failure in the identification of the top of the femoral head (for length measurements) in seven legs, the femoral head center (for angle measurements) in five legs, the placement of the distal femoral knee joint orientation line in one leg, and the proximal tibial knee joint orientation line in one leg. This led to the exclusion of the following LAMA angle measurements: two HKA, five mLDFA, and one mMPTA measurement in six LLRs. With regard to length measurements by LAMA, seven femur lengths, seven FLLs, and seven LLDs (22 of 29 pairs of legs had correct landmark placements in both legs) were omitted. Landmark placement was correct in 91% to 98% of the cases with regard to angle measurements and in 76 to 100% with regard to length measurements (Table 2). 

### 3.1. Comparison of Manual Observations 

One observer (an advanced practitioner in radiography) reported slightly higher mean values for HKA and mLDFA, while the second observer (a pediatric orthopedic surgeon) measured higher means for LLD. However, the differences between both observers for all measurement variables were small (and considered clinically not to be relevant) and statistically non-significant. Moderate to excellent agreement between the observers for all measurement variables was observed (Table 3). 

### 3.2. Comparison of LAMA with Manual LLR Measurements

After the exclusion of measurements with erroneously placed landmarks, the LAMA software showed comparable mean values for all lengths and angles compared to the mean manual measurements. For length measurements, the agreement was excellent (ICC ≥ 0.99). For angle measurements, the ICC ranged from moderate to excellent agreement (ICC 0.73 (95%CI 0.54 to 0.84) to 0.97 (95%CI 0.70 to 0.99)) (Table 4). Based on inspection of the Bland–Altman plots, the difference in femur length measurements seemed to increase with larger femoral length (Bland–Altman Figure S1, see Supplementary Material). For the mMPTA, the difference between LAMA and manual measurements seemed to increase when the angle became smaller (Bland–Altman Figure S5, see Supplementary Material). 

## 4. Discussion

LLRs are frequently performed on children to assess and follow up on leg alignment and length differences, as well as for surgical planning. However, performing length and angle measurements on these radiographs is labor-intensive and time-consuming. To our knowledge, this was the first study in pediatric patients to assess the performance of automatically analyzed LLRs using a DL-based LAMA software application compared to manually annotated LLRs by two observers. We found that LAMA was able to accurately identify the anatomical landmarks that are needed for length and angle measurements in the vast majority of cases. However, because correct landmark placement ranged between 76% and 100% for different LLR measurements, LAMA should not be used in clinical practice to analyze the LLR of pediatric patients without oversight of landmark placement by a clinician. In cases where landmark placement was correct, the agreement between LLR measurements obtained with LAMA and manual measurements was high, as depicted in the ICCs for both length and angle measurements. 

Considering the existing literature on LAMA, Schwarz et al. reported correct landmark placement in 92% and produced an output rate (angle measurements) of 96% [16]. Simon et al. found an overall accurate landmark placement in 89% of cases and a higher output rate (length and angle measurements) of 98.0% [17]. Although we obtained an output rate in all of the cases with LAMA, the percentage of correctly placed anatomical landmarks in our study was slightly lower compared with these studies in adult patients. One explanation could be that ossification is still ongoing in (younger) children or that children with the indication for LLR have anatomical abnormalities (i.e., LLD or varus/valgus due to an underlying disease), making it difficult for LAMA to identify the right landmarks and draw the correct lines. Based on some of the observed erroneous landmark placements in our study by LAMA on the proximal tibial knee joint orientation line, it may seem that it can be difficult to find the most distal point in the tibia plateau groove in children (as depicted in Figure 2b). Also, for the human eye, finding the exact most distal central point of a reasonably flat surface on a 2D image can be rather difficult. Thus, resulting in more variability between human observers and LAMA. The rate of correct landmark placement may be further improved by providing data for the retraining of skeletally immature patients.

Length measurements with LAMA resulted in high output rates and landmark placements, except for LLD. As shown, correct landmark placement was considerably lower in cases of LLD measurements. To obtain the LLD, two correctly measured FLLs of a patient are needed (i.e., from both legs), requiring four correct landmarks for each measurement. When a single landmark required for FLL in one leg is incorrectly placed, LLD cannot be determined, thus explaining the somewhat higher exclusion rate for LLD measurements in our study. Also, the top of the femur head is one of the landmarks that is needed to calculate FLL and LLD. The presence of a deformed femoral head in children who have been identified as needing an LLR may hamper the correct identification of the top of the femur head landmark by LAMA. 

With regard to angle measurements, specifically, mMPTA measurements appeared difficult in our dataset, as reflected by lower ICC compared to other measurements. This finding does not correspond with Archer et al., who evaluated the agreement in LLD and knee alignment measurements between LAMA AI software and two manual observers in adult patients [20]. The latter study reported an ICC of 0.89 (95%CI 0.85 to 0.92) for mMPTA when comparing the output of an AI model with manual observers. Another study by Erne et al. on adult patients, using an algorithm based on AI for automated leg measurements on LLR, also showed a higher ICC for mMPTA (ICC > 0.83) between the AI model and manual measurements compared with our study [27]. 

A recent study conducted by Zheng et al. investigated a different DL-based model on an LLR dataset of children and found a high consistency of LLD measurements between automated DL-based and manual measurements (Pearson correlation coefficient (r) 0.94) [28]. Although r and ICC evaluate validity and reliability, they highlight different aspects, hampering a direct comparison of their results to the findings of our study. Whereas r assesses a linear relationship, ICC provides an absolute and more robust agreement between the two methods. Lastly, de Villeneuve et al. compared an algorithm based on a machine learning process with 11 orthopedic surgeons and found mean differences for mLDFA of 2.1°, MPTA 1.6°, and HKA 1.3° [29]. In our study, similar differences between manual and LAMA measurements were found.

Our results suggest that AI applications like LAMA have the potential to enhance the time efficiency of LLR assessment in the pediatric orthopedic setting. This could be a significant gain in high-volume clinics, as it reduces the time and effort that is required for leg length and joint angle measurements. Furthermore, improved efficiency and reduced variability could potentially lead to better patient outcomes by enabling early and accurate detection of deviating growth in pediatric patients. As for imaging, the precision of measurements may be further improved by using Cone–Beam Computed Tomography (CBCT) or low-dose biplanar digital X-ray systems [30], hereby eliminating factors such as rotation and fan effect distortion [31]. 

There are some limitations of our study to take into account. Firstly, given that the measurements performed by the senior pediatric orthopedic surgeon and advanced practitioner in radiography are not deemed flawless, the question arises as to whether measurement discrepancies can be attributed to the flawed measurements of LAMA or variability and inaccuracy of the observers (i.e., there is no gold standard). To assess the reliability of LAMA fully, it is important to underline that both inter- and intraobserver variability exist in manual observations. Whereas automated software applications, such as LAMA, will always provide the exact same measurements (i.e., there is no intraobserver variability), intraobserver variability will be present for manual observers. We found moderate to excellent interobserver agreement for all length and angle measurements on LLRs in our cohort of children, but we did not assess the intraobserver variability within the observers. However, the possible intraobserver variability within the manual observers is expected to be very small [4,32]. Therefore, we do not expect that the latter would have significantly influenced our findings with regard to the agreement between LAMA and manually performed measurements. Another limitation is that the reason for landmark misplacement by LAMA is not always clear due to the opaque nature of the software application. In our study, a case was observed where the hip joint anatomy was abnormal, and LAMA completely missed this landmark. In the latter example, it may be clear why the placement of a specific landmark went wrong, but in other cases without obvious osseous or other structural deformations or image artifacts, it may not always be clear why the placement of specific landmarks was performed erroneously. The use of explainable AI methods could help to understand the reason for incorrect landmark placement and indicate what could be done to improve landmark placement by the addition of specific data for retraining the model. Lastly, our sample size was relatively small, and we collected patients from a single center, which may limit the generalizability of our findings. 

## 5. Conclusions

In conclusion, our findings suggest that the LAMA software is a reliable tool for LLR measurements in a pediatric setting that can potentially save valuable time for the treating physician. The LAMA software application demonstrated correct landmark placement in 91% to 98% of cases with regard to angle measurements and in 76 to 100% with regard to length measurements. The latter underlines that manual oversight of landmark placement by the LAMA software in LLRs of pediatric patients is important. If the landmark placement was correct, high agreement of LAMA with manually performed measurements on LLRs was observed. 

## Figures and Tables

**Table 1:** Overview of landmarks and measurement variables.

Landmarks		Description	
1. Top of the femoral head		Most superior point of the femoral head	
2. Medial femoral condyle		Most distal point of the medial femoral condyle	
3. Mid-tibial roof		Middle of tibial plafond in the tibiotalar joint	
4. Mechanical axis of the tibia		Axis passing through the center of the ankle joint and the midpoint of the knee joint	
5. Proximal tibial knee joint orientation line		Line crossing the two lowest points of the tibia plateau	
6. Mechanical axis of the femur		Axis passing through the center of the femoral head and the midpoint of the knee joint	
7. Distal femoral knee joint orientation line		Line passing through the most distal points of the femoral condyles	
**Measurement Variables**	**Description**		**Landmarks Used**
Femur length	Distance between the most superior point of the femoral head and the most distal point of the medial femur condyle		1 and 2
Tibia length	Distance between the most distal point of the medial femoral condyle and mid-tibial roof		2 and 3
Full leg length	Distance between the most superior point of the femoral head and mid-tibial roof		1 and 3
Leg length discrepancy	Difference between the full leg lengths of both legs within the same patient		1 and 3
mMPTA	Angle between the mechanical tibial axis and proximal tibial knee joint orientation line		4 and 5
mLDFA	Angle between the mechanical femoral axis and the distal femoral joint orientation line		6 and 7
HKA	Angle between the mechanical femoral and tibial axes		6 and 4

mMPTA, mechanical medial proximal tibial angle; mLDFA, mechanical lateral distal femoral angle; HKA, hip–knee–ankle angle.

**Table 2:** Overview of correct landmark placements with AI software per measurement variable.

Measurement Variables	cLMP (%)	Legs Analyzed (n)
Femur length	88%	51
Tibia length	100%	58
FLL	88%	51
LLD	76%	44
mMPTA	98%	57
mLDFA	91%	53
HKA	97%	56

cLMP, correct landmark placement; FLL, full leg length; LLD, leg length discrepancy; mMPTA, mechanical medial proximal tibial angle; mLDFA, mechanical lateral distal femoral angle; HKA, hip–knee–ankle angle.

**Table 3:** Mean measurement and interobserver agreement of manual measurements on long leg radiographs (LLRs).

Measurements	Legs Analyzed (n)	Observer 1 (AP)	Observer 2 (OS)	Mean Difference	ICC
Femur length [mm]	51	512.2 (47.6)	512.2 (47.8)	0.0 (−0.4 to 0.5)	1.00 (1.00 to 1.00)
Tibia length [mm]	58	410.6 (43.3)	410.5 (43.3)	0.1 (−0.1 to 0.4)	1.00 (1.00 to 1.00)
FLL [mm]	51	923.5 (89.7)	923.5 (89.6)	0.0 (−0.5 to 0.4)	1.00 (1.00 to 1.00)
LLD [mm]	44	8.9 (9.2)	9.1 (9.2)	−0.2 (−0.8 to 0.3)	0.99 (0.98 to 1.00)
mMPTA [°]	57	88.6 (2.0)	88.5 (1.9)	0.1 (−0.2 to 0.4)	0.88 (0.79 to 0.93)
mLDFA [°]	53	87.0 (1.9)	86.4 (2.0)	0.8 (0.5 to 1.0)	0.91 (0.62 to 0.97)
HKA [°]	56	−0.2 (2.5)	−0.3 (2.5)	0.2 (0.0 to 0.3)	0.98 (0.97 to 0.99)

Measurements of both observers are presented as mean with standard deviation. Mean differences and intraclass correlation coefficients (ICCs) are presented with a 95% confidence interval. Lengths are presented in millimeters and angles in degrees. AP, advanced practitioner in radiography; OS, orthopedic surgeon; FLL, full leg length; LLD, leg length discrepancy; mMPTA, mechanical medial proximal tibial angle; mLDFA, mechanical lateral distal femoral angle; HKA, hip–knee–ankle angle.

**Table 4:** Comparison of mean long leg radiograph (LLR) measurements performed by two observers with the measurements obtained by the leg angle measurement assistant (LAMA).

Measurements	Legs Analyzed (n)	LAMA	Manual	Mean Difference	ICC
Femur length [mm]	51	511.3 (47.3)	512.2 (47.7)	0.9 (0.6 to 1.2)	1.00 (1.00 to 1.00)
Tibia length [mm]	58	411.4 (43.6)	410.6 (43.3)	−0.9 (−1.1 to −0.6)	1.00 (1.00 to 1.00)
FLL [mm]	51	923.8 (89.8)	923.5 (89.7)	−0.3 (−0.7 to 0.1)	1.00 (1.00 to 1.00)
LLD [mm]	44	9.0 (9.4)	9.0 (9.1)	0.0 (−0.5 to 0.4)	0.99 (0.99 to 1.00)
mMPTA [°]	57	87.4 (2.8)	88.5 (1.8)	1.2 (0.7 to 1.6)	0.73 (0.54 to 0.84)
mLDFA [°]	53	87.1 (2.2)	86.8 (1.9)	−0.3 (−0.6 to 0.0)	0.91 (0.84 to 0.95)
HKA [°]	56	0.3 (2.5)	−0.3 (2.5)	−0.6 (−0.8 to −0.4)	0.97 (0.82 to 0.99)

Measurements obtained with LAMA or manually are presented as mean with standard deviation. Mean differences and ICCs are presented with a 95% confidence interval. Lengths are presented in millimeters and angles in degrees. The mean values of the measurements of the two observers are compared to the measurements obtained with the leg angle measurement assistant (LAMA). Lengths are presented in millimeters and angles in degrees. FLL, full leg length; LLD, leg length discrepancy; mMPTA, mechanical medial proximal tibial angle; mLDFA, mechanical lateral distal femoral angle; HKA, hip–knee–ankle angle.

## Data Availability

The original contributions presented in the study are included in the article/Supplementary Materials; further inquiries can be directed to the corresponding author.

## Supplementary Materials

The following supporting information can be downloaded at: https://www.mdpi.com/article/10.3390/children11101182/s1, Figure S1: Bland-Altman plot of the artificial intelligence (AI) and manual femur length measurements. Figure S2: Bland-Altman plot of the artificial intelligence (AI) and manual tibia length measurements. Figure S3: Bland-Altman plot of the artificial intelligence (AI) and manual FLL measurements. Figure S4: Bland-Altman plot of the artificial intelligence (AI) and manual LLD measurements. Figure S5: Bland-Altman plot of the artificial intelligence (AI) and manual mMPTA measurements. Figure S6: Bland-Altman plot of the artificial intelligence (AI) and manual mLDFA measurements. Figure S7: Bland-Altman plot of the artificial intelligence (AI) and manual HKA measurements.
