# Supplementary material for: Automated Measurements of Long Leg Radiographs in Pediatric Patients: A Pilot Study to Evaluate an Artificial Intelligence-Based Algorithm
Source: Children (Basel). 2024 Sep 27;11(10):1182. doi: 10.3390/children11101182 (PMC11505924; doi:10.3390/children11101182)
Supplement: Supplementary file 1 [file children-11-01182-s001.zip › children-3209443-supplementary.pdf]

Supplementary Material

1. **Femur length**

Figure S1. Bland-Altman plot of the artificial intelligence (AI) and manual femur length measurements.

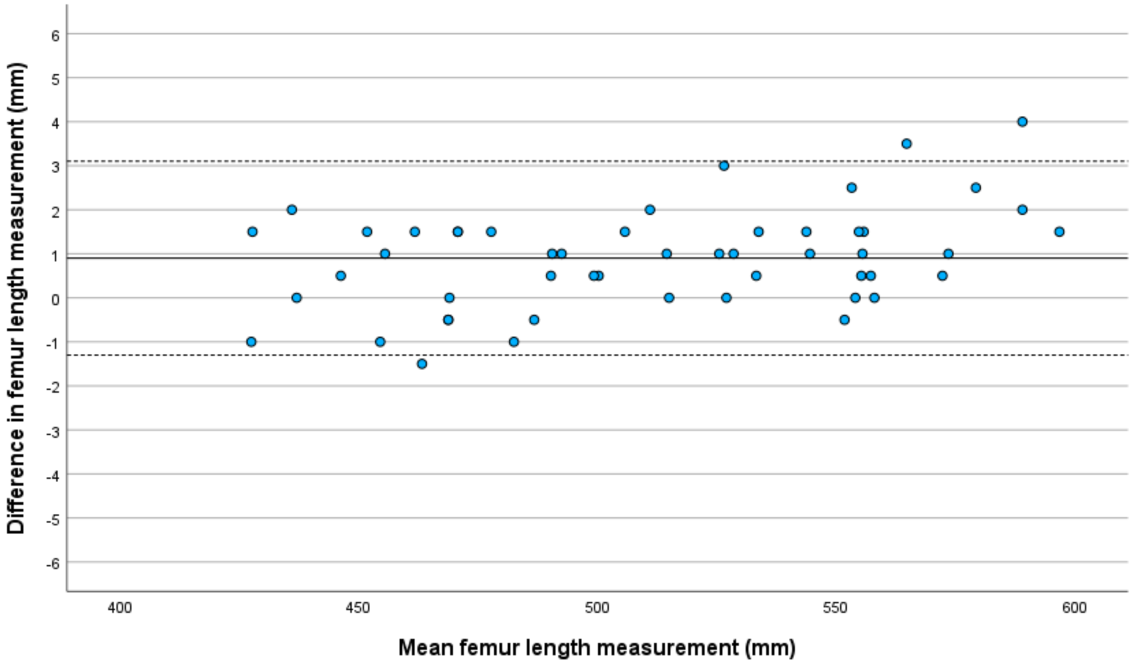

The solid line horizontal line represents the mean of differences. The dashed horizontal lines show the 95% limits of agreement.

2. **Tibia length**

Figure S2. Bland-Altman plot of the artificial intelligence (AI) and manual tibia length measurements.

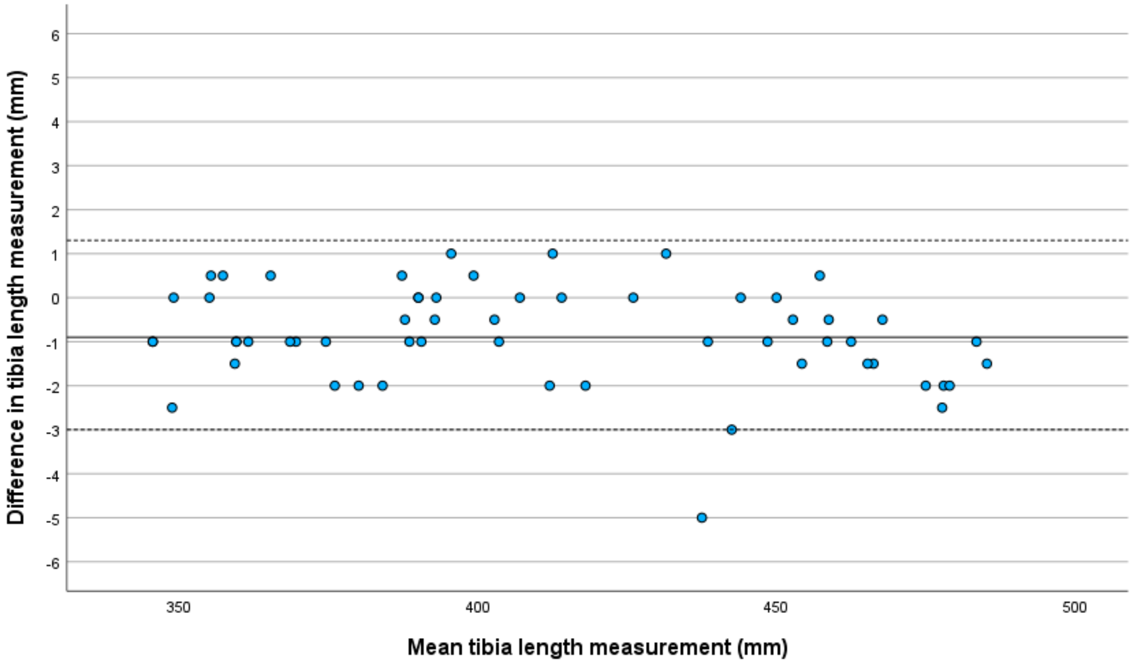

The solid line horizontal line represents the mean of differences. The dashed horizontal lines show the 95% limits of agreement.

3. **Full leg length (FLL)**

Figure S3. Bland-Altman plot of the artificial intelligence (AI) and manual FLL measurements.

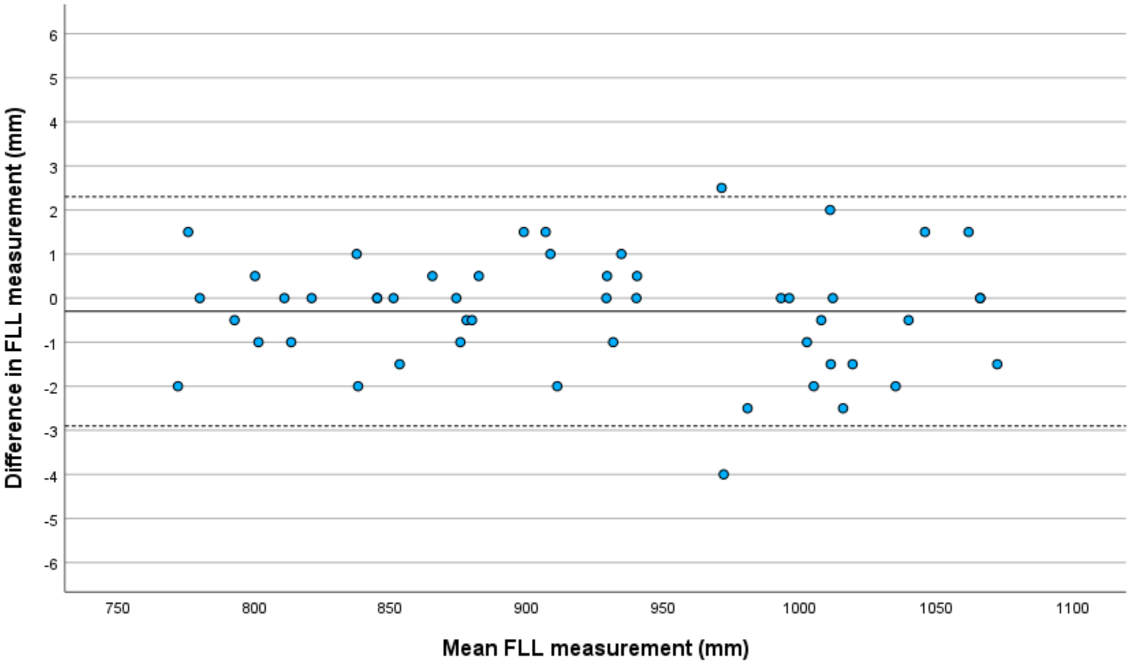

The solid line horizontal line represents the mean of differences. The dashed horizontal lines show the 95% limits of agreement.

4. **Leg length discrepancy (LLD)**

Figure S4. Bland-Altman plot of the artificial intelligence (AI) and manual LLD measurements.

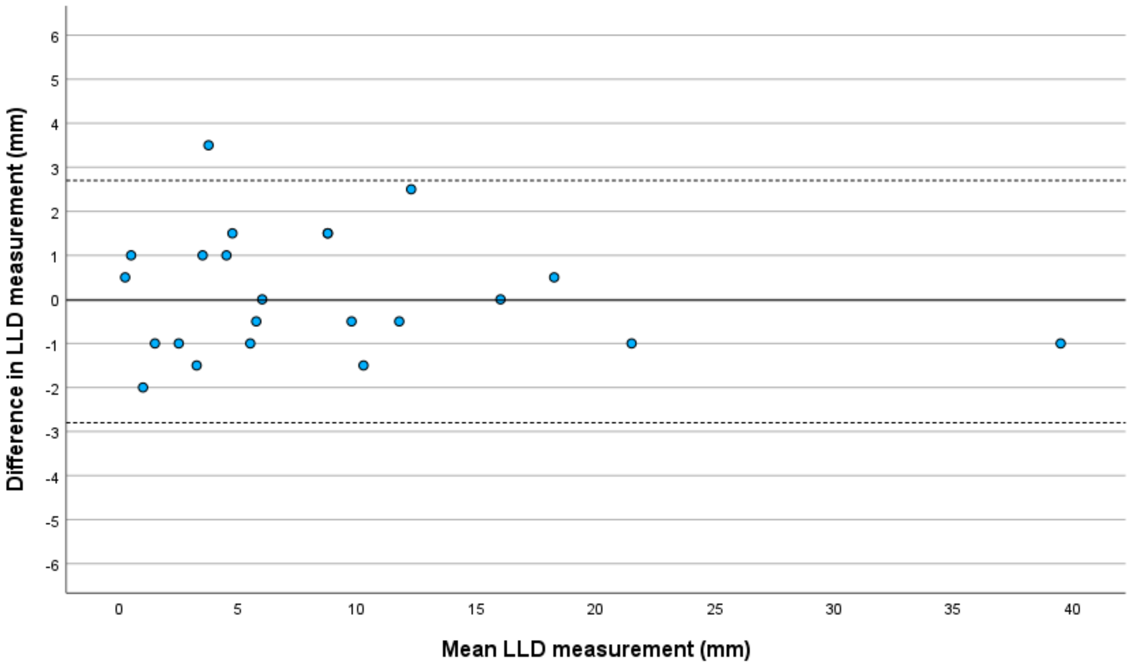

The solid line horizontal line represents the mean of differences. The dashed horizontal lines show the 95% limits of agreement.

5. Mechanical medial proximal tibial angle (mMPTA)

Figure S5. Bland-Altman plot of the artificial intelligence (AI) and manual mMPTA measurements.

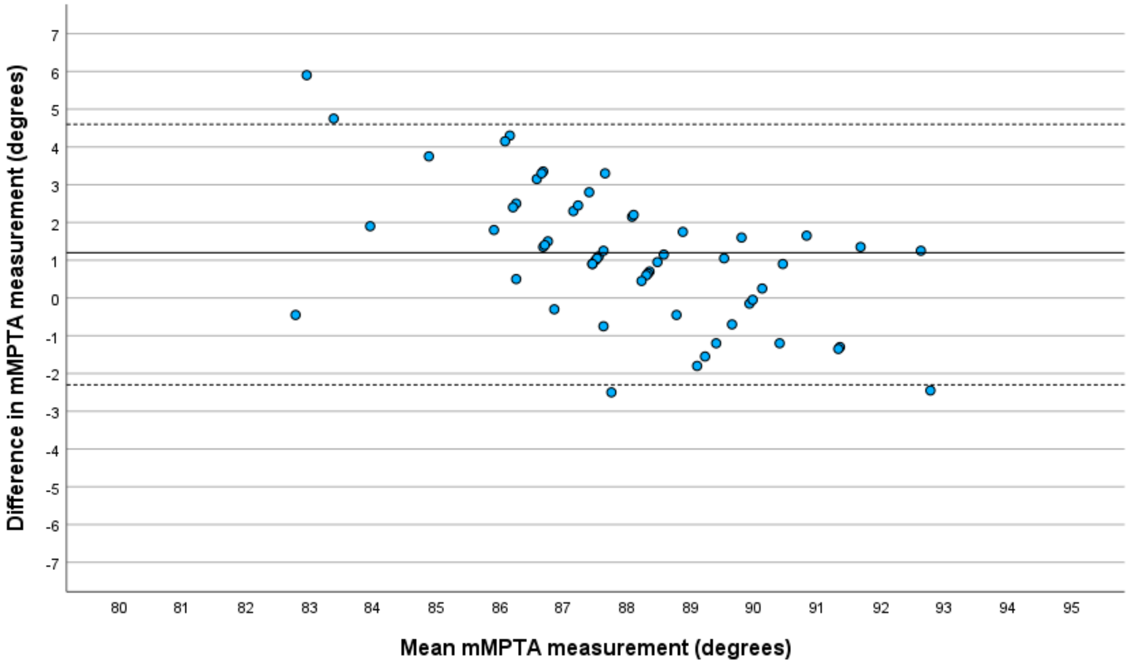

The solid line horizontal line represents the mean of differences. The dashed horizontal lines show the 95% limits of agreement.

6. Mechanical lateral distal femoral angle (mLDFA)

Figure S6. Bland-Altman plot of the artificial intelligence (AI) and manual mLDFA measurements.

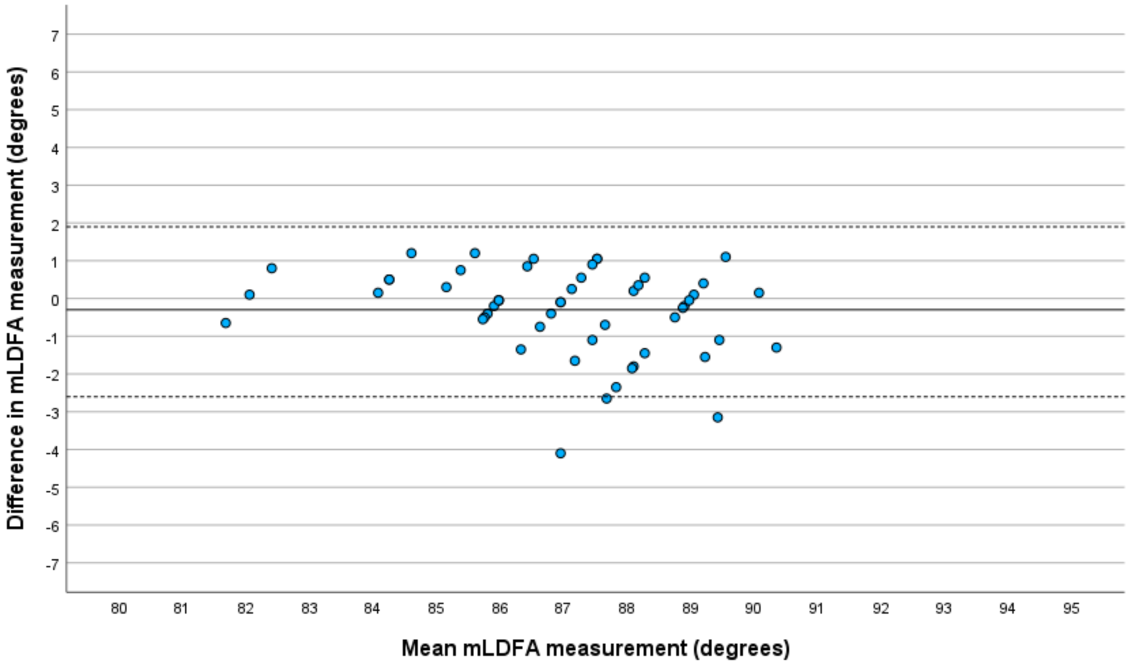

The solid line horizontal line represents the mean of differences. The dashed horizontal lines show the 95% limits of agreement.

7. **Hip-knee-ankle angle (HKA)**

Figure S7. Bland-Altman plot of the artificial intelligence (AI) and manual HKA measurements.

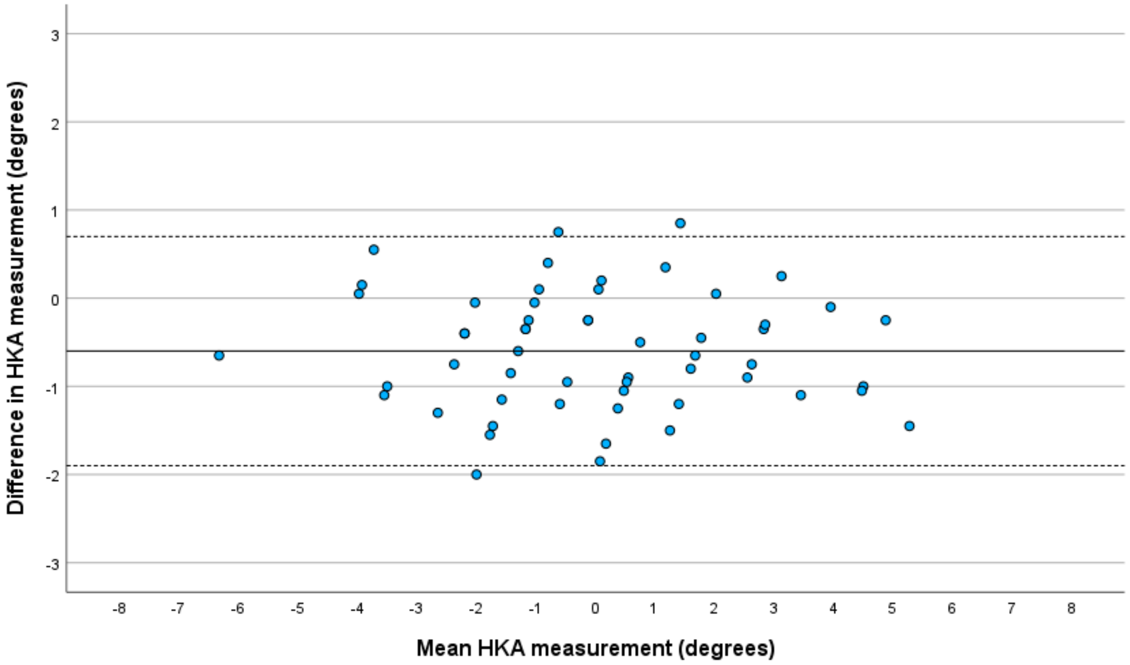

The solid line horizontal line represents the mean of differences. The dashed horizontal lines show the 95% limits of agreement.
